# Neuroanatomical Variability and Substance Use Initiation in Late Childhood and Early Adolescence

**DOI:** 10.1001/jamanetworkopen.2024.52027

**Published:** 2024-12-30

**Authors:** Alex P. Miller, David A. A. Baranger, Sarah E. Paul, Hugh Garavan, Scott Mackey, Susan F. Tapert, Kimberly H. LeBlanc, Arpana Agrawal, Ryan Bogdan

**Affiliations:** 1 Department of Psychiatry, Indiana University School of Medicine, Indianapolis; 2 Department of Psychological and Brain Sciences, Washington University in St Louis, Missouri; 3 Department of Psychiatry, University of Vermont Larner College of Medicine, Burlington; 4 Department of Psychiatry, University of California, San Diego; 5 Division of Extramural Research, National Institute on Drug Abuse, Bethesda, Maryland; 6 Department of Psychiatry, Washington University in St Louis School of Medicine, St Louis, Missouri

## Abstract

**Importance:**

The extent to which neuroanatomical variability associated with early substance involvement, which is associated with subsequent risk for substance use disorder development, reflects preexisting risk and/or consequences of substance exposure remains poorly understood.

**Objective:**

To examine neuroanatomical features associated with early substance use initiation and to what extent associations may reflect preexisting vulnerability.

**Design, Setting, and Participants:**

Cohort study using data from baseline through 3-year follow-up assessments of the ongoing longitudinal Adolescent Brain Cognitive Development Study. Children aged 9 to 11 years at baseline were recruited from 22 sites across the US between June 1, 2016, and October 15, 2018. Data were analyzed from February to September 2024.

**Exposures:**

Substance use initiation through 3-year follow-up (ie, age <15 years).

**Main Outcomes and Measures:**

Self-reported alcohol, nicotine, cannabis, and other substance use initiation and baseline magnetic resonance imaging (MRI)–derived estimates of brain structure (ie, global and regional cortical volume, thickness, surface area, sulcal depth, and subcortical volume). Covariates included family (eg, familial relationships), pregnancy (eg, prenatal exposure to substances), child (eg, sex and pubertal status), and MRI (eg, scanner model) variables.

**Results:**

Among 9804 children (mean [SD] baseline age, 9.9 [0.6] years; 5160 boys [52.6%]; 213 Asian [2.2%], 1474 Black [15.0%], 514 Hispanic/Latino [5.2%], 29 American Indian [0.3%], 10 Pacific Islander [0.1%], 7463 White [76.1%], and 75 other [0.7%]) with nonmissing baseline neuroimaging and covariate data, 3460 (35.3%) reported substance use initiation before age 15. Initiation of any substance or alcohol use was associated with thinner cortex in prefrontal regions (eg, rostral middle frontal gyrus, β = −0.03; 95% CI, −0.02 to −0.05; *P* = 6.99 × 10^−6^) but thicker cortex in all other lobes, larger globus pallidus and hippocampal volumes, as well as greater global indices of brain structure (eg, larger whole brain volume, β = 0.05; 95% CI, 0.03 to 0.06; *P* = 2.80 × 10^−8^) following Bonferroni or false discovery rate multiple testing correction. Cannabis use initiation was associated with lower right caudate volume (β = −0.03; 95% CI, −0.01 to −0.05; *P* = .002). Post hoc examinations restricting to postbaseline initiation suggested that the majority of associations, including thinner prefrontal cortex and greater whole brain volume, preceded initiation.

**Conclusions and Relevance:**

In this cohort study of children, preexisting neuroanatomical variability was associated with substance use initiation. In addition to putative neurotoxic effects of substance exposure, brain structure variability may reflect predispositional risk for initiating substance use earlier in life with potential cascading implications for development of later problems.

## Key Points

**Question:**

What are the neuroanatomical features associated with early substance use initiation, and do they precede initiation?

**Findings:**

In this cohort study of 9804 participants, variability in brain structure, including greater whole brain, cortical, and subcortical volumes, and thinner prefrontal cortex, but thicker cortex otherwise, was significantly associated with early (ie, age <15 years) substance use initiation (ie, alcohol, nicotine, cannabis, or other). The majority of brain structure features associated with substance use initiation were evident among substance-naive children at baseline who later initiated.

**Meaning:**

These findings suggest neurodevelopmental variability in brain structure may confer risk for early substance involvement.

## Teaser

This cohort study evaluates neuroanatomical features associated with early substance use initiation and whether these associations reflect preexisting risk of developing substance use disorders.

## Introduction

The widespread prevalence and devastating consequences of substance use constitute an international public health concern.^1^ While the adverse outcomes associated with substance use typically emerge in late adolescence and/or young adulthood, they are often set in motion earlier. As earlier onset of substance use is a potent predictor of later problematic use,^2,3,4,5^ it is critical to understand risk factors and mechanisms contributing to initial substance experimentation.

Neuroimaging studies have revealed that substance involvement (eg, initiation, use, escalating or problematic use, or substance use disorder [SUD]) is associated with lower gray matter volume, thinner cortex, and less white matter integrity.^6,7,8,9,10^ While these patterns are often observed globally, some associations are regionally potentiated (eg, hippocampus^11,12,13^ or dorsolateral prefrontal cortex [dlPFC]^6,7,14^). With few exceptions,^15,16,17,18^ these findings have emerged from cross-sectional studies and are largely speculated to be substance-induced.^19,20,21,22^

Some longitudinal magnetic resonance imaging (MRI) studies are consistent with substance-induced brain differences^6,9^; however, converging evidence from longitudinal and genetically informed studies suggests that these differences may also reflect predisposing risk for substance involvement arising from genetic and environmental exposures.^7,14,15,18,23^ For instance, lower volume and thinner dlPFC of substance-naive children predicts future alcohol involvement,^7,10,18^ family history of alcohol use disorder is associated with thinner frontal cortices in substance-naive youths,^24^ and low-drinking twin and nontwin siblings of high-drinking individuals have similarly smaller dlPFC and insula volumes as their heavy drinking counterparts.^7^ Similar associations have been observed for other substances (eg, cannabis).^23,25^ These findings suggest that neuroanatomical correlates of substance involvement may, at least partially, reflect predisposing risk factors, in addition to direct consequences of use. This broadly aligns with genetically informed models hypothesizing that neurobiological correlates of addiction may reflect both causes and consequences,^26^ as well as with developmental theories (eg, dual-systems or maturational imbalance models) highlighting brain development as a predispositional factor for substance involvement.^27,28^

We examined associations between brain structure and substance use initiation in children enrolled in the ongoing Adolescent Brain and Cognitive Development (ABCD) Study.^29^ We first tested whether baseline neuroanatomical variability was associated with any substance use initiation occurring before or up to 3 years following initial neuroimaging scans. We then tested whether any observed associations were present when excluding children who reported substance use initiation before the baseline neuroimaging session (ie, in a substance-naive subsample). Evidence that neuroanatomical variability in substance-naive youths predicts future substance use initiation would suggest that these correlates may represent predispositional risk factors for substance involvement. While neuroanatomical features associated with substance involvement are largely shared across different substances, substance-specific effects have also been found.^8^ Thus, we also examined neuroanatomical correlates of the 3 most commonly used substances in early adolescence: alcohol, nicotine, and cannabis. Broadly, we hypothesized that neuroanatomical features associated with substance use initiation would be evident even before use began, consistent with their conceptualization as putative predispositional risk factors. Specifically, we hypothesized that substance use initiation would be characterized by smaller global neuroanatomical indices, and that these differences would be particularly pronounced in regions previously identified as putative predispositional risk markers (ie, dlPFC and insula).

## Methods

### Participants

The ABCD Study^29^ is a longitudinal study of complex behavior and biological development from middle childhood to young adulthood. A total of 11 875 children aged 8.9 to 11 years at baseline (born 2005-2009) were recruited from 22 US research sites. Parents or caregivers provided written informed consent and children provided assent to a research protocol approved by the institutional review board at each site.^30^ Race and ethnicity were reported by parent or caregiver and were assessed to characterize the sociodemographic variability of our sample. Baseline neuroimaging data were drawn from ABCD data release 3.0; all other data were drawn from release 5.0. Final analytic samples after missing data exclusion (eMethods in Supplement 2) were 6556 to 9804 participants (Table). This study followed Strengthening the Reporting of Observational Studies in Epidemiology (STROBE) reporting guidelines.

**Table.** Sample Demographics by Substance Use Initiation Endorsement in Current Analytic Adolescent Brain Cognitive Development Sample by 3-Year Follow-Up

Characteristic	Participants, No. (%)^a^				
	Any (n = 3460)^b^	Alcohol (n = 3123)	Nicotine (n = 431)	Cannabis (n = 212)	Substance-naive (n = 6344)
Endorsing use at baseline	2257 (65.2)	2190 (70.1)	109 (25.3)	11 (5.2)	NA
Sex					
Male	1942 (56.1)	1754 (56.2)	238 (55.2)	129 (60.8)	3218 (50.7)
Female	1518 (43.9)	1369 (43.8)	193 (44.8)	83 (39.2)	3126 (49.3)
Baseline age, mean (SD), y	10.00 (0.63)	10.00 (0.63)	10.12 (0.61)	10.17 (0.61)	9.87 (0.62)
Race and ethnicity^c^					
Asian	59 (1.7)	56 (1.8)	2 (0.5)	2 (1.0)	154 (2.4)
American Indian/Alaska Native	11 (0.3)	9 (0.3)	5 (1.2)	1 (0.5)	18 (0.3)
Black/African American	362 (10.5)	285 (9.1)	57 (13.3)	42 (20.0)	1112 (17.6)
Hispanic/Latino	143 (4.1)	116 (3.7)	23 (5.4)	26 (12.4)	371 (5.9)
Pacific Islander	1 (<0.1)	1 (<0.1)	0	0	9 (0.1)
White	2852 (82.6)	2629 (84.3)	339 (79.0)	137 (65.2)	4611 (72.9)
Other	26 (0.8)	23 (0.7)	3 (0.7)	2 (1.0)	49 (0.8)
Household income, US $					
<25 000	328 (10.3)	264 (9.1)	76 (19.6)	51 (26.4)	897 (15.4)
25 000-49 999	430 (13.5)	358 (12.4)	87 (22.4)	43 (22.3)	889 (15.2)
50 000-74 999	417 (13.0)	363 (12.5)	79 (20.4)	34 (17.6)	847 (14.5)
75 000-99 999	452 (14.1)	413 (14.3)	46 (11.9)	21 (10.9)	887 (15.2)
100 000-199 999	1089 (34.1)	1033 (35.7)	65 (16.8)	33 (17.1)	1762 (30.2)
≥200 000	481 (15.1)	465 (16.1)	35 (9.0)	11 (5.7)	562 (9.6)
Median range	75 000-99 999	100 000-199 999	50 000-74 999	50 000-74 999	75 000-99 999
Baseline pubertal status					
Prepubertal	1769 (51.1)	1626 (52.1)	167 (38.7)	87 (41.0)	3280 (51.7)
Early pubertal	857 (24.8)	773 (24.8)	104 (24.1)	59 (27.8)	1464 (23.1)
Midpubertal	777 (22.5)	678 (21.7)	148 (34.3)	58 (27.4)	1503 (23.7)
Late pubertal	52 (1.5)	42 (1.3)	11 (2.6)	7 (3.3)	90 (1.4)
Postpubertal	5 (0.1)	4 (0.1)	1 (0.2)	1 (0.5)	7 (0.1)
Prenatal substance exposure^d^					
Any	1464 (44.9)	1336 (45.5)	214 (52.2)	102 (50.3)	1798 (29.9)
Alcohol	1176 (36.4)	1094 (37.5)	134 (33.3)	63 (31.3)	1287 (21.4)
Nicotine	521 (15.5)	446 (14.7)	133 (32.1)	58 (28.0)	769 (12.4)
Cannabis	245 (7.3)	211 (7.0)	57 (13.9)	34 (16.4)	309 (5.0)

Abbreviation: NA, not applicable.

^a^ Participant data were missing for the following variables: race and ethnicity (26 participants), household income (763 participants), pubertal status (90 participants), and prenatal exposure (524 participants). χ^2^ Tests revealed that substance initiating and substance-naive participants did not differ in missing data.

^b^ Includes 213 participants endorsing recreational use of substances other than alcohol, nicotine, and cannabis, such as inhalants (66 participants), stimulants (32 participants), synthetic cannabis/salvia (32 participants), prescription sedatives (31 participants), over the counter cough or cold medicine (27 participants), opioids (12 participants), hallucinogens (6 participants), or other (6 participants).

^c^ Child race and ethnicity were reported by parents or caregivers at baseline. Other race and ethnicity includes other race reported, declined to respond, or unknown.

^d^ Prenatal substance exposure includes maternal substance use before or after knowledge of pregnancy. Any includes alcohol, nicotine, cannabis, or other substances (ie, cocaine, opioids, methamphetamine, or other drugs).

### Substance Use Initiation

Annual in-person (ie, baseline and follow-ups at 1 year, 2 years, and 3 years) and midyear phone interviews (at 6 months, 18 months, and 30 months) assessed substance use. Participants endorsing use at any assessment from baseline to follow-up 3 (ie, lifetime use) were included in substance use initiation groups. Alcohol (808 participants) and nicotine (34 participants) use endorsed solely in the context of religious ceremonies were coded as missing to restrict comparisons to use outside these settings.

Alcohol use initiation (3123 participants) was defined as sipping or full drinks of alcohol (eMethods in Supplement 2). Nicotine use initiation (431 participants) was defined as use of nicotine products in any form or route of administration. Cannabis use initiation (212 participants) was defined as use of cannabis in any form or route of administration except synthetic cannabis or cannabis-infused alcoholic drinks, which were included under any substance use. Any substance use initiation (3460 participants) was defined as alcohol, nicotine, or cannabis use initiation or any other illicit substance use (213 participants). Substance-naive participants (6344 participants) endorsed no substance use from baseline to follow-up 3 with nonmissing data for follow-up 3 to protect against misclassification of participants whose follow-up 3 status was unknown (1065 participants). eTable 1 in Supplement 1 lists study release variables.

### Structural Magnetic Resonance Imaging

Baseline structural MRI data, obtained via 3T MRI scanners and harmonized across 3 MRI vendor platforms, were available for 11 556 of 11 875 participants following FreeSurfer processing and quality control protocols (eMethods in Supplement 2). In total, 297 imaging-derived phenotypes (IDPs) were examined in the current study, including global IDPs (ie, whole brain volume, total intracranial volume, total cortical volume, total cortical surface area, mean cortical thickness, mean cortical sulcal depth, and total subcortical gray matter volume; *k* = 7); bilateral volume of 9 subcortical structures (ie, accumbens, amygdala, caudate, cerebellar cortex, cerebellar white matter, globus pallidus, hippocampus, putamen, and thalamus; total *k* = 18); and total volume, mean thickness, total surface area, and mean sulcal depth of 34 bilateral cortical regions according to the Desikan-Killiany atlas^31^ (total *k* = 68/metric).

### Statistical Analysis

A series of independent linear mixed-effect regressions wherein IDPs were regressed on substance use initiation groups were conducted using the lme4 version 1.1-30 package in R version 4.2.1 (R Project for Statistical Computing).^32,33^ Variables were *z*-scored before analyses, and listwise deletion was used for missing data. Primary analyses contrasted any substance use initiation (3460 participants) vs no initiation (6344 participants); secondary analyses considered alcohol (3123 participants), nicotine (431 participants), and cannabis (212 participants) use initiation independently and contrasted them against no substance initiation (6344 participants). Fixed-effect covariates in all analyses were baseline age and age-squared, sex, pubertal status, familial relationship (ie, sibling, twin, or triplet), and MRI scanner model (eTable 2 in Supplement 1; eMethods and eFigure 1 in Supplement 2). Random intercepts were modeled for effects of family nested within data collection sites (eMethods in Supplement 2). For regional brain association models, the global metric was also included as a covariate (eg, mean cortical thickness for regional thickness) (eMethods in Supplement 2). Although sociodemographic characteristics are associated with both neuroanatomical development and substance use outcomes,^34,35,36,37,38,39^ these variables were not included as covariates as they may plausibly influence observed associations in meaningful ways, in which case, exploring these as putative mechanisms contributing to associations (a research question beyond the scope of the current study) would be more appropriate than controlling for them.

Multiple testing corrections were applied first using Bonferroni correction to all tests conducted as part of the study to prioritize especially robust results (*P* = .05/1188 = 4.21 × 10^−5^). Additional 5% false discovery rate (FDR) corrections were applied across all tests examining associations between IDPs and any substance use initiation (primary analysis; *k* = 297 tests), and all secondary tests of associations between IDPs and alcohol, nicotine, and cannabis use initiation (*k* = 891 tests), to highlight results not surviving the more stringent Bonferroni correction (eMethods in Supplement 2).

#### Post Hoc Analyses

As prenatal substance exposure is associated with substance use initiation^40^ and brain structure,^41,42^ we conducted post hoc analyses of FDR-significant associations controlling for prenatal substance exposures (eMethods in Supplement 2). Additionally, as a large portion of participants initiated substance use before the baseline study session (2257 participants), we examined whether FDR-significant associations remained when restricting substance use initiation groups to only participants endorsing initiation following the baseline assessment (1203 participants for any substance; 933 participants for alcohol; 322 participants for nicotine; and 201 participants for cannabis) to test whether IDP correlates were present before initiation (eMethods in Supplement 2). Finally, both restriction of analytic samples to postbaseline substance use initiation and inclusion of prenatal exposure as a covariate were incorporated in post hoc tests to examine associations that might reflect predispositional variability independent of prenatal substance exposure. Data were analyzed from February to September 2024.

## Results

Among our full analytic sample of 9804 participants (5160 boys [52.6%]; mean [SD] baseline age, 9.9 [0.6] years; 213 Asian [2.2%], 1474 Black [15.0%], 514 Hispanic/Latino [5.2%], 29 American Indian [0.3%], 10 Pacific Islander [0.1%], 7463 White [76.1%], and 75 reporting other or unknown race or declining to respond [0.7%]), 3460 ABCD participants initiated substance use before age 15 with 3123 (90.2%) of these reporting alcohol use initiation and considerable overlap among initiation of alcohol, nicotine, and cannabis use (Table, eResults, eFigure 2 in Supplement 2).

**Figure 1.**
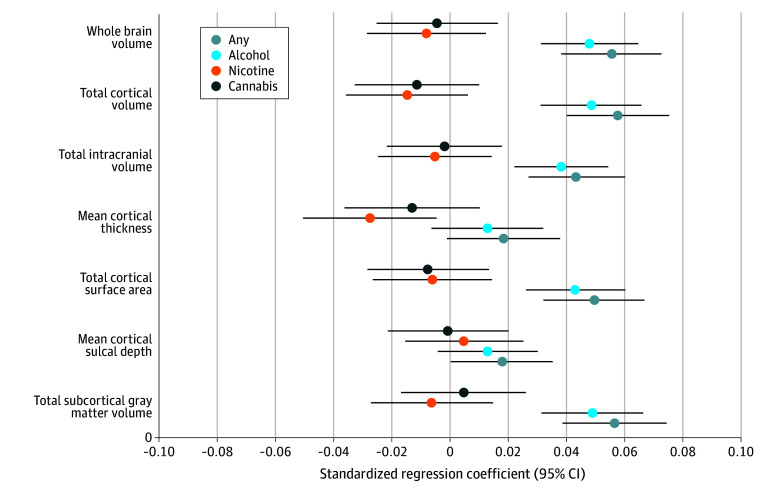
Global Brain Metric Associations With Early Substance Use Initiation in the Adolescent Brain Cognitive Development (ABCD) Study Standardized regression coefficients with 95% CIs for associations between global metrics and substance use initiation.

**Figure 2.**
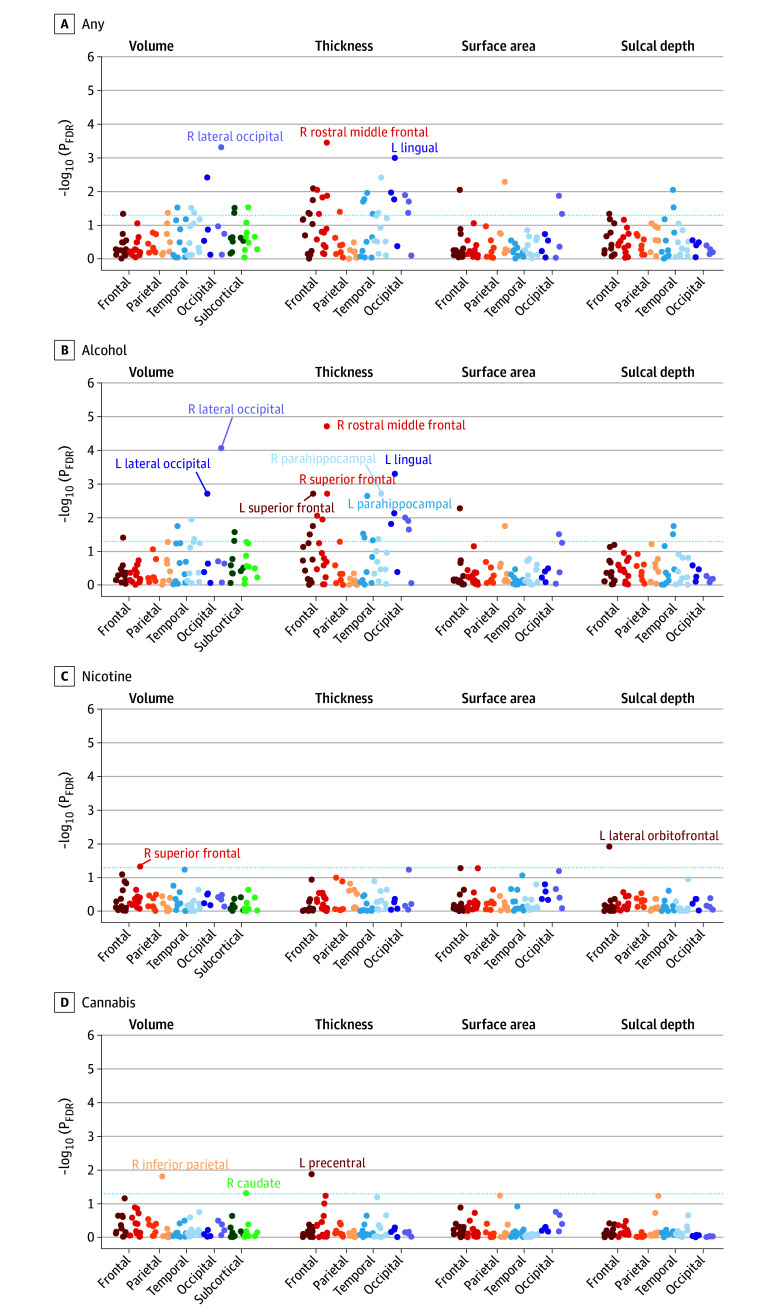
Manhattan Plots of Regional Neuroanatomical Associations With Early Substance Use Initiation in the Adolescent Brain Cognitive Development (ABCD) Study This figure plots –log_10_-transformed false discovery rate (FDR)–corrected *P* values (*P*_FDR_) from mixed-effect regressions for all regional association analyses grouped within neuroanatomical metrics for each substance (ie, cortical and subcortical volume, thickness, surface area, and sulcal depth). *P* values are aggregated and color coded by cortical lobes and subcortical regions with darker colors reflecting left (L) hemisphere and lighter colors reflecting right (R) hemisphere for each region (eg, dark red indicates L frontal lobe and lighter red indicates R frontal lobe). Although often considered separate from frontal, parietal, and temporal lobes, and located at their junction, for simplicity the insular cortex is plotted here along with temporal regions. Dashed blue line reflects *P*_FDR_ < .05. For any substance and alcohol use (A, B), labeled regions reflect associations that are Bonferroni-significant for all study comparisons (*P* < .05 / 1188=4.21 × 10^−5^). For nicotine and cannabis use (C, D), labeled regions reflect FDR-significant associations.

**Figure 3.**
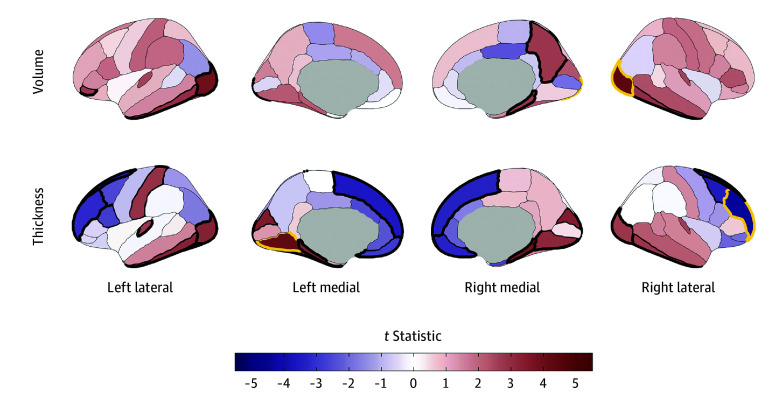
Regional Cortical Volume and Thickness Associations With Early Substance Use Initiation in the Adolescent Brain Cognitive Development (ABCD) Study Cortical volume and thickness patterning of associations with any substance use initiation are plotted as *t *statistics (red indicates a positive association and blue indicates a negative association) according to the Desikan–Killiany cortical atlas.^31^ Regions with bold yellow outlines were Bonferroni-significant for all study comparisons, and those with bold black outlines exhibited false discovery rate (FDR)–significant associations (eTable 3 in Supplement 1). Regional brain plots were constructed using the ggseg package in R.^43^

Eight IDPs were associated with any early substance use initiation after Bonferroni correction (eTables 3-4 in Supplement 1). Any substance use initiation was associated with larger global neuroanatomical indices (*k* = 5), including whole brain (β = 0.05; 95% CI, 0.03 to 0.06; *P* = 2.80 × 10^−8^), total intracranial (β = 0.04; 95% CI, 0.02 to 0.05; *P* = 3.49 × 10^−6^), cortical (β = 0.05; 95% CI, 0.03 to 0.07; *P* = 4.31 × 10^−8^), and subcortical volumes (β = 0.05; 95% CI, 0.03 to 0.07; *P* = 4.39 × 10^−8^), as well as greater total cortical surface area (β = 0.04; 95% CI, 0.03 to 0.06; *P* = 6.05 × 10^−7^) (Figure 1). Regionally (*k* = 3), thinner right rostral middle frontal gyrus (β = −0.03; 95% CI, −0.02 to −0.05; *P* = 6.99 × 10^−6^), thicker left lingual gyrus (β = 0.03; 95% CI, 0.02 to 0.05; *P* = 2.65 × 10^−5^), and larger right lateral occipital gyrus volume (β = 0.04; 95% CI, 0.02 to 0.05; *P* = 1.12 × 10^−5^) were associated with any substance use initiation (Figure 2A and Figure 3).^43^ An additional 36 regional IDPs were also associated with any substance use initiation using FDR correction (Figure 2A, Figure 3; eFigure 3 in Supplement 2; eTable 3 in Supplement 1). Of all regional associations (ie, 3 Bonferroni-significant plus 36 FDR-significant associations; *k* = 39), the majority were with cortical thickness (22 of 39 [56.4%]). Notably, any substance use initiation was characterized by thinner cortex in all frontal regions (*k* = 9), but thicker cortex in all other lobes (occipital *k* = 6, parietal *k* = 1, and temporal *k* = 6). Any substance use initiation was also associated with larger regional brain volumes (subcortical *k* = 3, and cortical *k* = 7), deeper regional sulci (*k* = 3), and differences in regional cortical surface area (*k* = 4).

**Figure 4.**
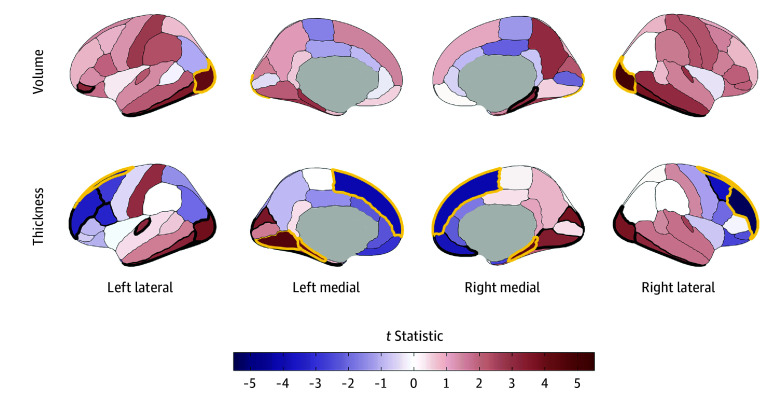
Regional Cortical Volume and Thickness Associations With Early Alcohol Use Initiation in the Adolescent Brain Cognitive Development (ABCD) Study Cortical volume and thickness patterning of associations with alcohol use initiation are plotted as *t* statistics (red indicates a positive association and blue indicates a negative association) according to the Desikan–Killiany cortical atlas.^31^ Regions with bold yellow outlines were Bonferroni-significant for all study comparisons, and those with bold black outlines exhibited false discovery rate (FDR)–significant associations (eTable 5 in Supplement 1). Regional brain plots were constructed using the ggseg package in R.^43^

Secondary analyses compared the 3 most used substances (alcohol, nicotine, and cannabis) with no substance use. Unsurprisingly, given the preponderance of alcohol use initiation in this sample, alcohol findings largely recapitulated those observed for any substance use. Thirteen associations with alcohol use initiation remained significant after Bonferroni correction. In addition to identifying the same global and regional IDPs that were associated with any substance use initiation described previously (*k* = 8), the following additional IDPs (*k* = 5) were also significant after Bonferroni correction: greater left lateral occipital volume (β = 0.04; 95% CI, 0.02 to 0.05; *P* = 2.57 x 10^−5^) and bilateral parahippocampal gyri cortical thickness (right, β = 0.04; 95% CI, 0.02 to 0.06; *P* = 2.52 x 10^−5^; left, β = 0.04; 95% CI, 0.02 to 0.06; *P* = 3.25 x 10^−5^) and less bilateral superior frontal gyri cortical thickness (right, β = −0.03; 95% CI, −0.02 to −0.04; *P* = 2.34 x 10^−5^; left, β = −0.03; 95% CI, −0.01 to −0.04; *P* = 2.47 x 10^−5^) (Figure 2B, Figure 4; eTables 5-6 in Supplement 1). Following FDR correction, nicotine use was associated with lower right superior frontal gyrus volume (β = −0.03; 95% CI, −0.01 to −0.05; *P* = .002) and deeper left lateral orbitofrontal cortex sulci (β = 0.05; 95% CI, 0.02 to 0.07; *P* = 2.68 x 10^−4^), and cannabis use was associated with thinner left precentral gyrus (β = −0.03; 95% CI, −0.01 to −0.05; *P* = 3.26 x 10^−4^) and lower right inferior parietal gyrus (β = −0.03; 95% CI, −0.02 to −0.05; *P* = 4.13 x 10^−4^) and right caudate volumes (β = −0.03; 95% CI, −0.01 to −0.05; *P* = .002) (Figure 2C-D; eFigure 4 in Supplement 2; eTables 7-10 in Supplement 1). These associations were not robust to Bonferroni correction.

Results were largely consistent in models including a separate random effect of site (eTable 11 in Supplement 1). All FDR- and Bonferroni-significant associations remained significant when including prenatal exposure as a covariate in post hoc analyses (eTables 3, 5, 7, and 9 in Supplement 1). Most Bonferroni-significant associations (14 of 21 [66.7%]) remained significant when removing participants who endorsed substance initiation before the baseline neuroimaging session, as well as most global (eg, whole brain) and subcortical (eg, globus pallidus) volume associations and many cortical thickness findings (eg, right rostral middle frontal gyrus for alcohol) (eTables 3 and 5 in Supplement 1). All FDR-significant results for nicotine and cannabis use initiation remained significant when excluding participants endorsing baseline nicotine and cannabis use (eTables 7 and 9 in Supplement 1). eTable 12 in Supplement 1 summarizes all post hoc analyses. All effect sizes in the full sample were highly correlated with those from the sample that excluded baseline initiation (median [IQR] *r*, 0.79 [0.76-0.85]) (eResults, eFigure 5 in Supplement 2).

## Discussion

Early substance use initiation is associated with escalating use, use of multiple substances, SUD development, and other adverse life outcomes (eg, lower educational attainment and elevated psychopathology).^44,45,46,47^ We identified neuroanatomical features associated with early substance use initiation (ie, being aged <15 years), most of which are evident before any substance exposure. The direction of association between cortical thickness and substance use initiation was regionally specific; the cortical mantle was thinner in prefrontal regions, but thicker in temporal, occipital, and parietal regions among youths who initiated substance use. While age is associated with cortical thickness broadly across brain regions,^48^ our data suggest that regionally specific differences, rather than global age-related trends, may confer vulnerability to substance use initiation. SUDs and in particular alcohol use disorder have been characterized by broad reductions in cortical thickness in multiple brain regions with largest effects found in the frontal cortex.^8,20^ That regional associations may precede substance use initiation, including less cortical thickness in the right rostral middle frontal gyrus, challenges predominant interpretations that these associations arise largely due to neurotoxic consequences of exposure^8,20,49^ and increases the plausibility that these features may, at least partially, reflect markers of predispositional risk.^15,26^

This finding has important implications for brain-based theoretical models of addiction. The stage-based neurobiological model of addiction speculates that predominantly substance-induced variability in frontal regions contributes to later stages of addiction wherein compulsive use and craving develop following repeated drug pairings and related disruption of prefrontal afferent regulation of subcortical reward and stress-related circuitry.^50^ Our findings suggest that structural differences in the prefrontal cortex may predispositionally contribute to initial stages of substance involvement. Neurodevelopmental theories postulate that typical asynchronous regional brain maturation (ie, rapid subcortical development and later prefrontal development) leaves adolescents vulnerable to substance involvement by promoting emotional saliency in the context of underdeveloped cognitive control.^51,52^ Our findings of thinner frontal cortex alongside larger subcortical volumes at baseline (mean [SD] age, 9.9 [0.6] years) being associated with early substance use initiation are challenging to interpret with respect to these theories. Total cortical thickness peaks at age 1.7 years and steadily declines throughout life with limited evidence of regionally specific trajectories.^53^ By contrast, subcortical volumes peak at 14.4 years of age and generally remain stable before steep later life declines.^53^ Our data cannot yet determine at what developmental point(s) substance-related variability in brain structure arose (eg, whether thinner prefrontal cortices are attributable to less thickness developing through infancy and/or arise from greater pruning in later childhood). Large-scale longitudinal studies (eg, the Healthy Brain and Child Development Study^54^) tracking neurodevelopment and substance exposure or involvement from the neonatal period to young adulthood are necessary to address the origins of these differences.

Unlike negative associations between whole and regional brain volumes with adult substance use and SUDs,^8,55,56^ and contrary to our hypotheses, we found that greater global and regional cortical volumes were associated with early substance use initiation; greater subcortical volumes and total surface area were as well. However, it is important to consider that, while related to subsequent stages of substance involvement, there are considerable phenotypic differences between initiation in youths and problematic use in adults that may be associated with brain feature dissimilarities. Such differences could plausibly reflect opposing effects of predisposition and exposure-related changes or differences in developmental trajectories. Associations observed in the current study may be capturing variability related to exploration and risk-taking that has variable impact on progression of use.^57^ Notably, larger globus pallidus volume, here associated with substance use initiation and in other ABCD studies associated with sensation seeking,^58^ has been linked to both occasional use and SUDs in adults,^20^ highlighting a plausibly stable risk feature from precocious use and experimentation to later problematic use. Moreover, greater cortical surface area has been shown to be genetically correlated with alcohol use and sensation seeking in independent samples^25,59^ and related to family history of substance problems in ABCD,^60^ supporting the interpretation that greater surface area may reflect the influence of preexisting risk factors. Importantly, our findings are not incompatible with brain volume and/or surface area declines that may arise from substance exposure and/or differences in later brain development conferring risk for substance involvement progression.

### Limitations

This study had limitations. First, while our study was sufficiently powered to detect expected small effects for any substance and alcohol use initiation,^61^ less frequently endorsed individual substances may not have had adequate power (ie, 80% power for |β| > 0.04 at *P* < .005 for nicotine and cannabis). Although small effects suggest that these findings are not clinically informative for an individual, they do inform and challenge current theoretical models of addiction. Currently, there is limited variability in substance use involvement within released ABCD Study data, which restricted our investigation to associations with substance use initiation. It is possible that later, more problematic substance involvement phenotypes or multivariate approaches (eg, whole brain network analysis^62^) would yield larger effects. Such multivariate models could serve as omnibus tests to reduce multiple testing burden with subsequent post hoc univariate testing.

Second, while additional ABCD Study neuroimaging scans are available, relatively few substance-naive participants at baseline initiated before the next scan (417 participants), precluding well-powered models examining neuroanatomical changes associated with substance use initiation (ie, potential exposure effects). As this sample continues to age and substance involvement becomes more common and variable, evaluating cooccurring trajectories of substance involvement and neuroanatomy will be valuable. Although such investigations cannot determine whether changes in neural phenotypes reflect a consequence of substance exposure or developmental predisposition that cooccurs, genetically informed study designs may be leveraged to test the plausibility of both.^15,26^

Third, most of the sample had complete data, missingness was not associated with primary outcomes, and we accounted for several known familial, pregnancy-related, and child-related confounding variables. However, unmeasured confounders and undetected systemic differences in missing data may have influenced associations. While sociodemographic, environmental, and genetic variables were not included as covariates, these are likely associated with both neuroanatomical variability and substance use initiation and may moderate associations between them. Furthermore, given substantial heritability estimates of neuroanatomy^63^ and evidence of family history of substance use problems as a risk factor for thinner frontal cortices,^24,64^ genetically informed studies are needed to examine genetic influences on substance-related neuroanatomical variability and evaluate the plausibility of environmental causality, including through possible sociodemographic differences.

## Conclusions

In this cohort study of 9804 children, we identified neuroanatomical features associated with substance use initiation that were present before substance exposure. Convergent with evidence from genetically informed (eg, discordant twin or sibling)^7,14,23^ and other longitudinal research,^7,10,18^ our data increasingly place interpretations that substance-related variability solely arises from substance exposure on a procrustean bed. Ultimately, a greater understanding of the links between brain structure and substance involvement may uncover predispositional risk factors that provide insight into the early causes of SUDs and clinically informative mechanisms through which myriad adverse health outcomes associated with substance involvement emerge.

## Notes

Supplement 1. **eTable 1. **ABCD Variables Used to Construct Substance Use Initiation Groups **eTable 2. **Generalized Linear Mixed-Effect Regression Model Output of Covariate Effects on Any Substance Use Initiation **eTable 3. **FDR-Significant Neuroanatomical Associations From Mixed-Effect Regressions Comparing Participants Reporting Any Substance Use Initiation vs Substance-Naive Participants **eTable 4. **All Neuroanatomical Associations From Mixed-Effect Regressions Comparing Participants Reporting Any Substance Use Initiation vs Substance-Naive Participants **eTable 5. **FDR-Significant Neuroanatomical Associations From Mixed-Effect Regressions Comparing Participants Reporting Alcohol Use Initiation vs Substance-Naive Participants **eTable 6. **All neuroanatomical Associations From Mixed-Effect Regressions Comparing Participants Reporting Alcohol Use Initiation vs Substance-Naive Participants **eTable 7. **FDR-Significant Neuroanatomical Associations From Mixed-Effect Regressions Comparing Participants Reporting Nicotine Use Initiation vs Substance-Naive Participants **eTable 8. **All Neuroanatomical Associations From Mixed-Effect Regressions Comparing Participants Reporting Nicotine Use Initiation vs Substance-Naive Participants **eTable 9. **FDR-Significant Neuroanatomical Associations From Mixed-Effect Regressions Comparing Participants Reporting Cannabis Use Initiation vs Substance-Naive Participants **eTable 10. **All Neuroanatomical Associations From Mixed-Effect Regressions Comparing Participants Reporting Cannabis Use Initiation vs Substance-Naive Participants **eTable 11. **All Neuroanatomical Associations From Mixed-Effect Regressions With a Separate Random Effect of Site Comparing Participants Reporting Any Substance, Alcohol, Nicotine, or Cannabis Use Initiation vs. Substance-Naive Participants **eTable 12. **Summary of Bonferroni and FDR-Significant Results Remaining Significant Following a Second FDR Correction After Post Hoc Tests

Supplement 2.
eMethods.

eResults.
**eFigure 1. **Sensitivity Analysis Comparing Effect Estimates From Pre- and Post-ComBat Harmonization **eFigure 2.** Alcohol, Nicotine, and Cannabis Use Initiation in Current Analytic ABCD Sample Through Follow-Up Year 3 **eFigure 3.** Regional Cortical and Subcortical Associations With Any Early Substance Use Initiation in ABCD **eFigure 4.** Regional Cortical and Subcortical Associations With Early Alcohol, Nicotine, and Cannabis Use Initiation in ABCD **eFigure 5.** Correlations Between Effect Sizes in Analyses in the Full Sample and Analyses Restricted to Participants Without Baseline Substance Use Initiation

Supplement 3.
Data Sharing Statement
