# Supplementary material for: Neuroanatomical Variability and Substance Use Initiation in Late Childhood and Early Adolescence
Source: JAMA Netw Open. 2024 Dec 30;7(12):e2452027. doi: 10.1001/jamanetworkopen.2024.52027 (PMC11686416; doi:10.1001/jamanetworkopen.2024.52027)
Supplement: Supplement 2. — eMethods. eResults. eFigure 1. Sensitivity Analysis Comparing Effect Estimates From Pre- and Post-ComBat Harmonization eFigure 2. Alcohol, Nicotine, and Cannabis Use Initiation in Current Analytic ABCD Sample Through Follow-Up Year 3 eFigure 3. Regional Cortical and Subcortical Associations With Any Early Substance Use Initiation in ABCD eFigure 4. Regional Cortical and Subcortical Associations With Early Alcohol, Nicotine, and Cannabis Use Initiation in ABCD eFigure 5. Correlations Between Effect Sizes in Analyses in the Full Sample and Analyses Restricted to Participants Without Baseline Substance Use Initiation [file jamanetwopen-e2452027-s002.pdf]

Supplemental Online Content

Miller AP, Baranger DAA, Paul SE, et al. Neuroanatomical variability and substance use initiation in late childhood and early adolescence. *JAMA Netw Open*. 2024;7(12):e2452027. doi:10.1001/jamanetworkopen.2024.52027

eMethods.
eResults.
eFigure 1. Sensitivity Analysis Comparing Effect Estimates From Pre- and Post-ComBat Harmonization
eFigure 2. Alcohol, Nicotine, and Cannabis Use Initiation in Current Analytic ABCD Sample Through Follow-Up Year 3
eFigure 3. Regional Cortical and Subcortical Associations With Any Early Substance Use Initiation in ABCD
eFigure 4. Regional Cortical and Subcortical Associations With Early Alcohol, Nicotine, and Cannabis Use Initiation in ABCD
eFigure 5. Correlations Between Effect Sizes in Analyses in the Full Sample and Analyses Restricted to Participants Without Baseline Substance Use Initiation

This supplemental material has been provided by the authors to give readers additional information about their work.

© 2024 Miler AP et al. *JAMA Network Open.*

eMethods.

Alcohol sipping

While more specific indices of quantity, frequency, and level of use are available for alcohol in current Adolescent Brain Cognitive Development (ABCD) Study data releases (e.g., 'sips' *vs.* 'full drinks', number of sipping occasions[1,2]), here we focused on broad-spectrum use *vs.* no use to provide more consistent variable definitions across substances. Additionally, prior work has shown that alcohol sipping at these ages in ABCD is consistently associated with impulsivity and other aspects of externalizing psychopathology, highlighting its relevance as an appropriate alcohol-related phenotype for children and early adolescents.[1,2]

Child race and ethnicity

Race and ethnicity, along with household income, were assessed to characterize the sociodemographic variability of our sample. Race was queried in the ABCD Parent Demographic Survey using the question, "What race do you consider the child to be?" Parents/caregivers were allowed to select from 16 racial categories: (1) White; (2) Black/African American; (3) American Indian/Native American; (4) Alaska Native; (5) Native Hawaiian; (6) Gaumanian; (7) Samoan; (8) Other Pacific Islander; (9) Asian Indian; (10) Chinese; (11) Filipino; (12) Japanese; (13) Korean; (14) Vietnamese; (15) Other Asian; (16) Other Race; (17) Refuse to Answer; and (18) Don't Know. In the same survey, parents/caregivers were also asked, "Do you consider the child Hispanic/Latino/Latina?" From these responses we formed dichotomous groups for the most prevalent categories of race: White (Category 1), Black/African American (Category 2), Native American/Alaska Native (Categories 3-4), Pacific Islander (Categories 5-8), and Asian (Categories 9-15). Remaining participants were assigned to Other (Categories 16-18). Hispanic ethnicity was also considered. Race and ethnicity variables were dummy coded as non-mutually exclusive dichotomous variables; as such, participants were coded within both race and ethnicity, separately.

Structural magnetic resonance imaging

Briefly, 1mm isotropic T1-weighted structural images were obtained via 3T MRI scanners (Siemens, Phillips, and GE) using either a 32- or 64-channel head-and-neck coil and completed T1-weighted and T2-weighted structural scans (1mm isotropic).[3] Structural MRI (sMRI) scan protocols were harmonized across the three MRI vendor platforms to minimize variability. Real-time motion detection and correction was implemented to mitigate the influence of head motion. sMRI processing was completed using FreeSurfer version 5.3.0 through standardized processing pipelines and described quality control procedures performed by the ABCD Study® Data Analysis, Informatics and Resources Center using the FreeSurfer image analysis suite (http://surfer.nmr.mgh.harvard.edu/).[4] Participants who did not pass FreeSurfer QC measures (i.e., at least one T1 scan that passed all QC metrics) were excluded from analyses. Cortical reconstruction and volumetric segmentation was performed by the ABCD Study® Data Analysis, Informatics and Resources Center using the FreeSurfer image analysis suite (http://surfer.nmr.mgh.harvard.edu/). This pre-processing includes removal of non-brain tissue using a hybrid watershed/surface deformation procedure,[5] automated Talairach transformation, segmentation of the subcortical white matter and deep gray matter volumetric structures, intensity normalization, tessellation of the gray/white matter boundary, automated topology correction, and surface deformation following intensity gradients.[4] Cortical images were registered to the Desikan-Killiany atlas, which was based on individual cortical folding patterns to match cortical geometry across participants. The cerebral cortex was parcellated into 34 regions per hemisphere based on the gyral and sulcal structure. For sulcal depth, regions that moved outward during inflation were positive and represent the depths of sulci, and regions that moved inward were negative and represent the height of gyri.[6] In addition to global metrics (e.g., total intracranial volume, mean cortical thickness) and volumes of subcortical structures, regional metrics included in analyses were cortical volume, thickness, surface area, and sulcal depth. While models examining individual differences in regional cortical thickness and surface area highlight nuances in cortical morphology not fully captured by regional volume, regional volume predominates prior sMRI research. Moreover, prior research looking at both cortical surface area and cortical thickness suggests that these metrics measure unique aspects of cortical morphology and show unique correlations with complex behavior.[7,8] As there is a multiplicative effect between cortical surface area, thickness, and sulcal depth in cortical volume, and because there are no strong priors for one metric *vs.* others with respect to neuroanatomical variability associated with substance use initiation, we chose to examine all, balancing increased multiple testing burden and false negatives given small effects with optimal resolution on especially robust associations with morphology.

© 2024 Miler AP et al. *JAMA Network Open.*

Fixed effect covariates

Variables that could confound associations were included as fixed effect covariates in all analyses: baseline age and baseline age-squared,[9,10] sex,[11,12] pubertal status,[13–15] familial relationship (i.e., sibling, twin, triplet),[16,17] and MRI scanner model.[18,19] For pubertal status, parents and children both completed a 5-item scale on the child's pubertal development,[20] combined to a summary score. The parent rating was used as the primary measure. Given high congruence between parent and child ratings reported previously,[21,22] the child rating (*n*=223) was used if the parent rating was unavailable to reduce data loss to missingness as done in previous ABCD studies.[23,24] Family variables were included as both a random effect (i.e., family ID within site) and as fixed effect covariates (e.g., familial relationship: sibling, twin, and triplet status). These variables were included to account for non-independence of data arising from shared variance within families, which may result in artificially reduced standard errors and inflated *P*-values. Specifically, random effect of family ID was used to account for neuroanatomical variability that is correlated within families and thus may differ between families, while specific familial relationships (*rel_relationship*), including sibling (*n*=1,794), twin (*n*=2,090 monozygotic/dizygotic twins), and triplet status (*n*=30), were used as fixed effects to account for relatedness structures within family not accounted for by family ID. To assess whether including MRI scanner model as a fixed effect covariate sufficiently accounted for nuisance effects, we conducted an additional sensitivity analysis of our primary outcome (i.e., any substance use initiation) after conducting ComBat data harmonization[25] with scanner model specified as our batch variable. Results of this sensitivity analysis demonstrated no changes in effect estimates after harmonizing sMRI data across scanner models (**eFigure 1**). Sociodemographic variables indexing socioeconomic status and social determinants of health are associated with both brain structure development and substance use outcomes.[26–31] These variables were not included as covariates as they may plausibly influence observed associations in meaningful ways, in which case exploring the mechanisms by which they might influence associations, a research question beyond the scope of the current study, would be more appropriate than controlling for them.

For models examining regional brain associations, global metrics were also included as covariates: total intracranial volume for regional cortical and subcortical structural volumes; total cortical surface area for regional surface area; mean cortical thickness for regional thickness; and mean sulcal depth for regional sulcal depth. For *post hoc* analyses controlling for prenatal exposure, baseline retrospective report of maternal substance use before or after knowledge of pregnancy, either specific to alcohol, nicotine, or cannabis, or any substance including other substances (i.e., cocaine, opioids, methamphetamine, or other drugs), was added to models as an additional fixed effect covariate. Significant effects following a second FDR correction (P_{FDR}<.05) for IDPs in *post hoc* analyses were interpreted to reflect predispositional variability associated with substance use initiation (restriction of analytic samples to post-baseline substance use initiation) and associations independent of prenatal substance exposure (inclusion of prenatal exposure as a covariate).

Output from a generalized linear mixed-effect regression model of covariate effects on any substance use initiation is included in **eTable 2 in Supplement 1.** Results of this model suggest significant associations between substance use initiation and age-squared (odds ratio [OR]=7.93; 95% CI, 1.28-49.01; *P*=.026), sex (male relative to female; OR=1.12; 95% CI, 1.06-1.19; $P=5.61\times10^{-5}$), sibling status (sibling in sample relative to no sibling; OR=0.86; 95% CI, 0.81-0.91, $P=3.77\times10^{-8}$), and scanner model 4 (relative to model 1; OR=0.79; 95% CI, 0.72-0.86; $P=1.35\times10^{-7}$). Similar effects for each of these covariates were observed in follow-up models of each variable predicting substance use initiation individually: age-squared (OR=1.27; 95% CI, 1.21-1.33; $P=3.44\times10^{-22}$), sex (male relative to female; OR=1.13; 95% CI, 1.08-1.19; $P=4.15\times10^{-7}$), sibling status (sibling in sample relative to no sibling; OR=0.86; 95% CI, 0.82-0.90, $P=7.32\times10^{-9}$), and scanner model 4 (relative to model 1; OR=0.76; 95% CI, 0.72-0.80; $P=3.50\times10^{-25}$).

Multiple testing correction

Much of the extant theoretical work on neurobiological mechanisms underlying substance involvement and substance use disorders and related structural neuroimaging research literature has focused on regional examinations of cortical and subcortical volume as well as other regional cortical metrics like thickness.[7,32–38] Thus, in the current study we chose to focus on both global (e.g., total intracranial volume) and regional neuroanatomical metrics (while controlling for global neuroanatomical metrics), to better align our research question with existing regional evidence and to allow for greater comparison of our findings both with prior ABCD studies[39–41] and this literature more broadly. However, as most of the structural neuroimaging literature on substance involvement has centered on later stages of substance involvement (i.e., substance use disorders) in adults and studies in children have been predominantly constrained to small samples that may provide poor priors, we sought to balance hypothesized associations with a hypothesis-free data-driven approach. We report two different corrections for multiple testing to balance false positive and false negative results. First, we applied Bonferroni correction to all tests conducted as part of our study (*k*=1,188) to protect against false positives and prioritize especially robust results. Second, we applied false discovery rate (FDR)

© 2024 Miler AP et al. *JAMA Network Open.*

correction to all tests of our primary comparison (i.e., any substance use initiation *vs.* substance naïve; k=297) and then to our subsequent substance-specific tests (i.e., alcohol, nicotine, cannabis use initiation; k=891) to limit the potential of false negative results while adjusting for multiple testing. As FDR does not assume independence between tests, its use may be overly liberal in the case of spatial autocorrelation between brain regions, resulting in a higher risk of false positive results, which may be less likely to replicate or generalize to other samples. However, Bonferroni correction of a large number of tests treated as independent may be overly conservative resulting in a greater risk of false negatives. For prenatal exposure and post-baseline initiation *post hoc* analyses, significant associations following a second FDR-correction (P_{FDR}<.05) were interpreted to reflect evidence that associations were independent of prenatal substance exposure and may plausibly reflect predispositional vulnerability, respectively.

© 2024 Miler AP et al. *JAMA Network Open.*

eResults.

Substance use initiation

A total of 3,460 (35.3%) ABCD participants in the current analytic sample endorsed initiation of substance use outside the context of religious ceremonies during at least one assessment, while 6,344 (64.7%) participants reported no substance use (substance naïve). The vast majority of those endorsing substance use initiation by FU3 had reported using alcohol (*n*=3,123; 90.2%) with fewer participants reporting use of nicotine (*n*=431; 12.5%) and cannabis (*n*=212; 6.1%), respectively (**Table 1**). Moreover, nearly all participants endorsing alcohol use initiation reported having 'sips' of alcohol (*n*=2,931; 94.2%) without endorsing 'full drinks' (*n*=192; 5.8%). Reported routes of administration of nicotine and cannabis varied across participants and assessments. In general, however, e-cigarettes or vaping were the predominant method of nicotine use with 76.5% of those reporting specific routes of administration endorsing e-cigarette use during at least one assessment and 61.5% endorsing e-cigarette use exclusively. Tobacco cigarette use was endorsed by 18.7% of those reporting specific routes of administration, while a similar proportion (22.5%) endorsed other forms of nicotine use (i.e., cigars, hookahs, pipes, chew, nicotine replacement patches). For cannabis, the most frequently endorsed specific route of administration was smoking flower (65.2%), followed by consuming edibles (48.9%), and using oils, concentrates, or tinctures (37.1%). Only 23.0% of participants endorsing specific routes of cannabis administration reported vaping flower, concentrates, or oils. Two-hundred and thirteen participants endorsed use of substances other than alcohol, nicotine, or cannabis (e.g., hallucinogens, stimulants, sedatives; see legend of **Table 1**) with more than 50% of these participants endorsing use of only these other substances.

There was considerable overlap among participants reporting use of alcohol, nicotine, and cannabis ($\chi^2(3)=251.35$, $P=3.33\times10^{-54}$; **eFigure 2A**). For instance, more than 65% of those endorsing nicotine or cannabis use also endorsed use of the other substance and/or alcohol, and 68 participants endorsed use of all three. As would be expected, cumulative endorsement of alcohol, nicotine, and cannabis use initiation increased from baseline to FU3 (**eFigure 2B**). However, while smaller subsets of participants endorsed use of nicotine (*n*=109) or cannabis (*n*=11) during the initial baseline assessment, more than 70% of those endorsing use of alcohol thus far in the study had done so at baseline (*n*=2,190; **Table 1**). Increasing rates of endorsement of cannabis (+1,927%) and nicotine (+395%) use initiation from baseline to FU3 outpaced increasing prevalence in alcohol use initiation (+147%; **eFigure 2B**). Notably, rates of prenatal substance exposure were consistently higher among participants in substance use initiation groups compared to substance-naïve participants with some evidence of substance specificity (i.e., alcohol, nicotine, and cannabis use initiation groups demonstrated the highest rates of prenatal alcohol, nicotine, and cannabis exposure, respectively; **Table 1**).

Comparison of effect sizes for full *vs.* restricted analytic samples

Global and regional effect sizes were correlated between analyses that included all participants and analyses that were restricted to only participants with no substance use initiation at the baseline visit (**eFigure 5**). As spatial auto-correlation prevents deriving a *P*-value directly from the correlation coefficient,[42] exchangeability block permutations with accelerated *P*-values were used to determine the significance of the correlations.[43,44] Analyses were restricted to unrelated participants in order to facilitate the identification of valid permutations of substance initiation variables that did not violate the hierarchical structure of the data (i.e., observations nested within families, nested within sites). All regression analyses were then re-run for 10,000 permutations of the substance initiation variables, and analyses then computed the correlation between effect sizes of the permuted data with the true effect sizes from analyses restricted to participants with no baseline substance use initiation. This then yields, for each correlation, 10,000 coefficients drawn from a valid null distribution (i.e., an empirical null distribution). A beta distribution was then fit to the empirical null distribution, and a *P*-value for each true correlation was computed.[45]

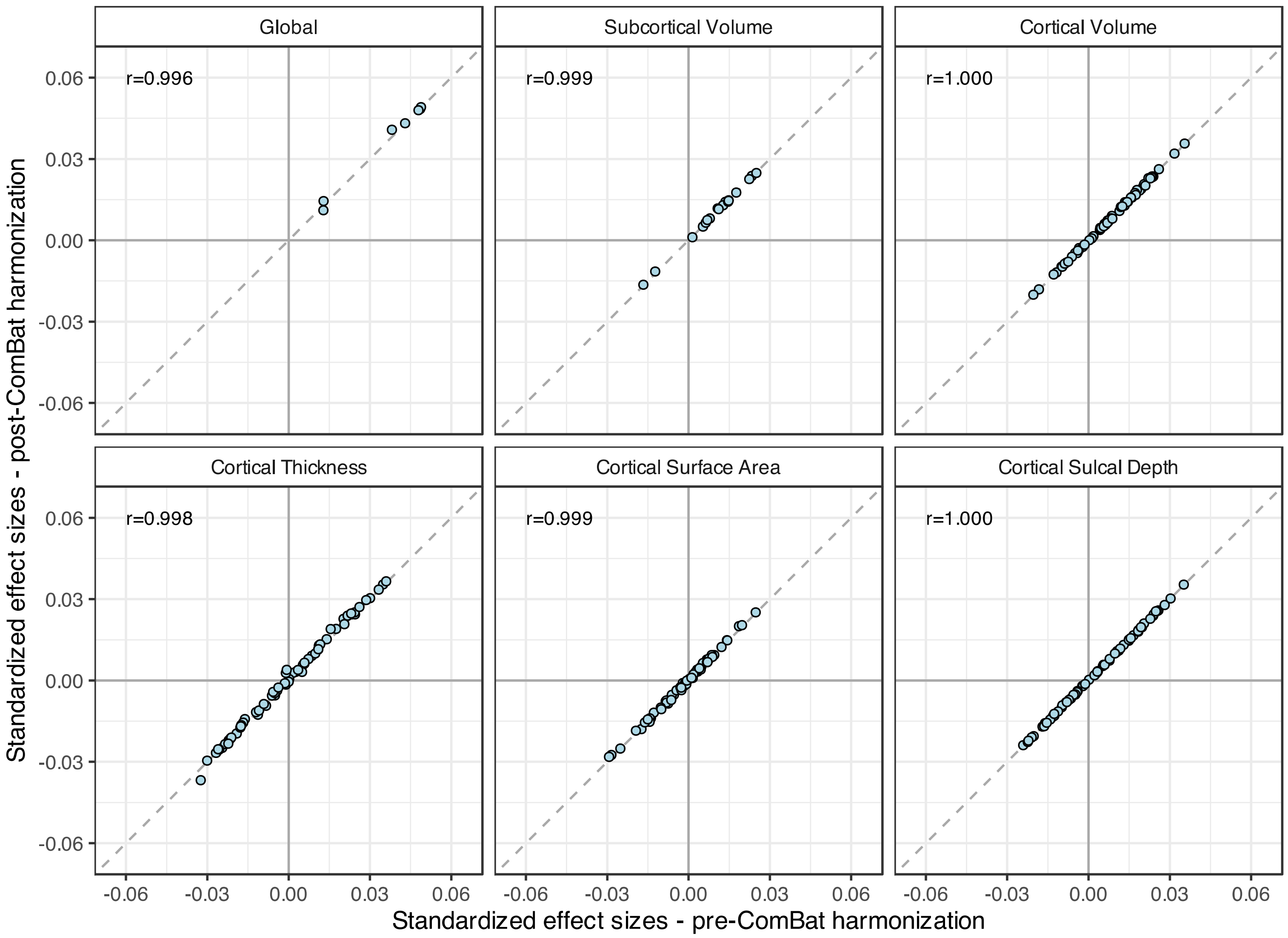

eFigure 1. Sensitivity analysis comparing effect estimates from pre- and post-ComBat harmonization. Comparison of effect estimates from primary outcome models (i.e., any substance use initiation) with MRI scanner model included as a fixed effect using unadjusted sMRI data (*x*-axis) *vs*. ComBat-harmonized sMRI data (*y*-axis).

© 2024 Miler AP et al. *JAMA Network Open.*

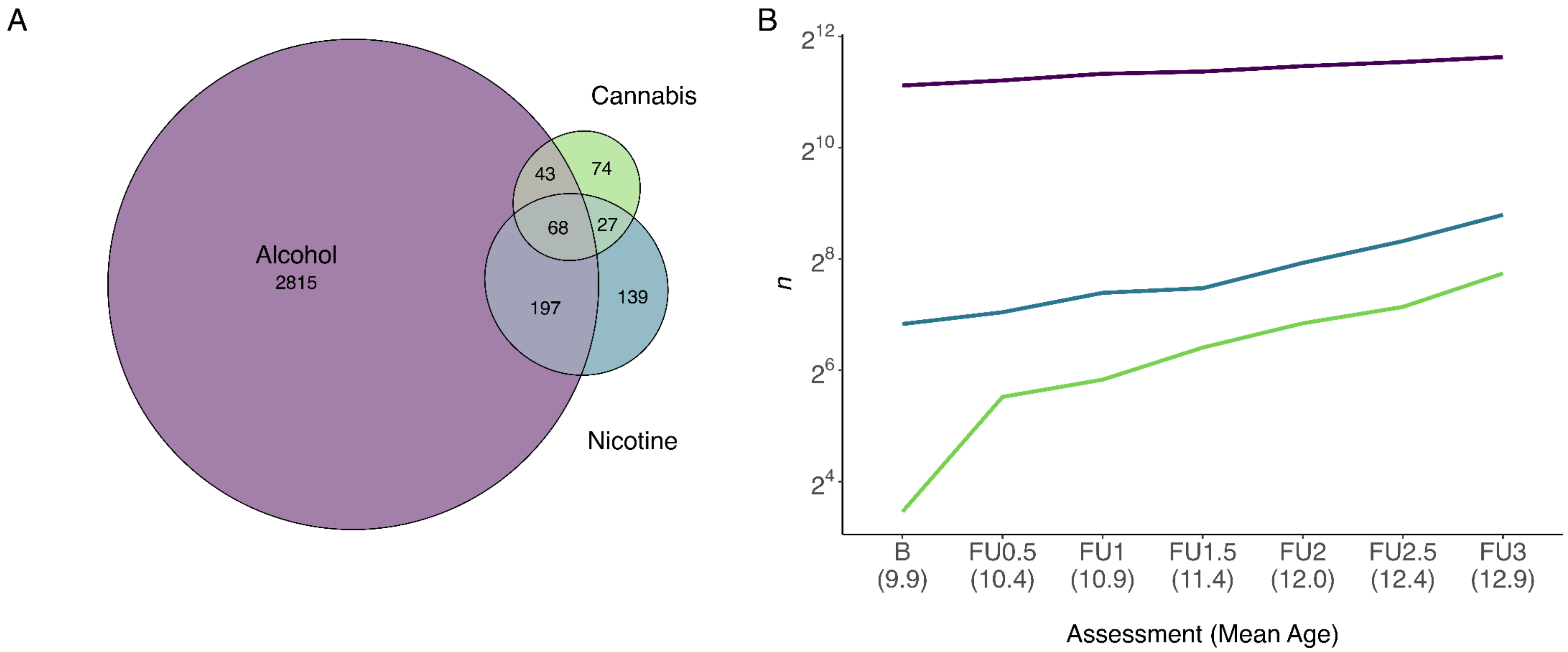

eFigure 2. Alcohol, nicotine, and cannabis use initiation in current analytic ABCD sample through follow-up year 3. A. Venn diagram exhibiting overlap amongst participants endorsing alcohol (including both 'sipping' and 'full drinks'), nicotine, and cannabis use initiation through follow-up year 3. **B.** Cumulative alcohol (including both 'sipping' and 'full drinks'), nicotine, and cannabis use initiation from baseline (mean age=9.9 years) to third follow-up assessment (mean age=12.9 years). *y*-axis log2-transformed sample size.

© 2024 Miler AP et al. *JAMA Network Open.*

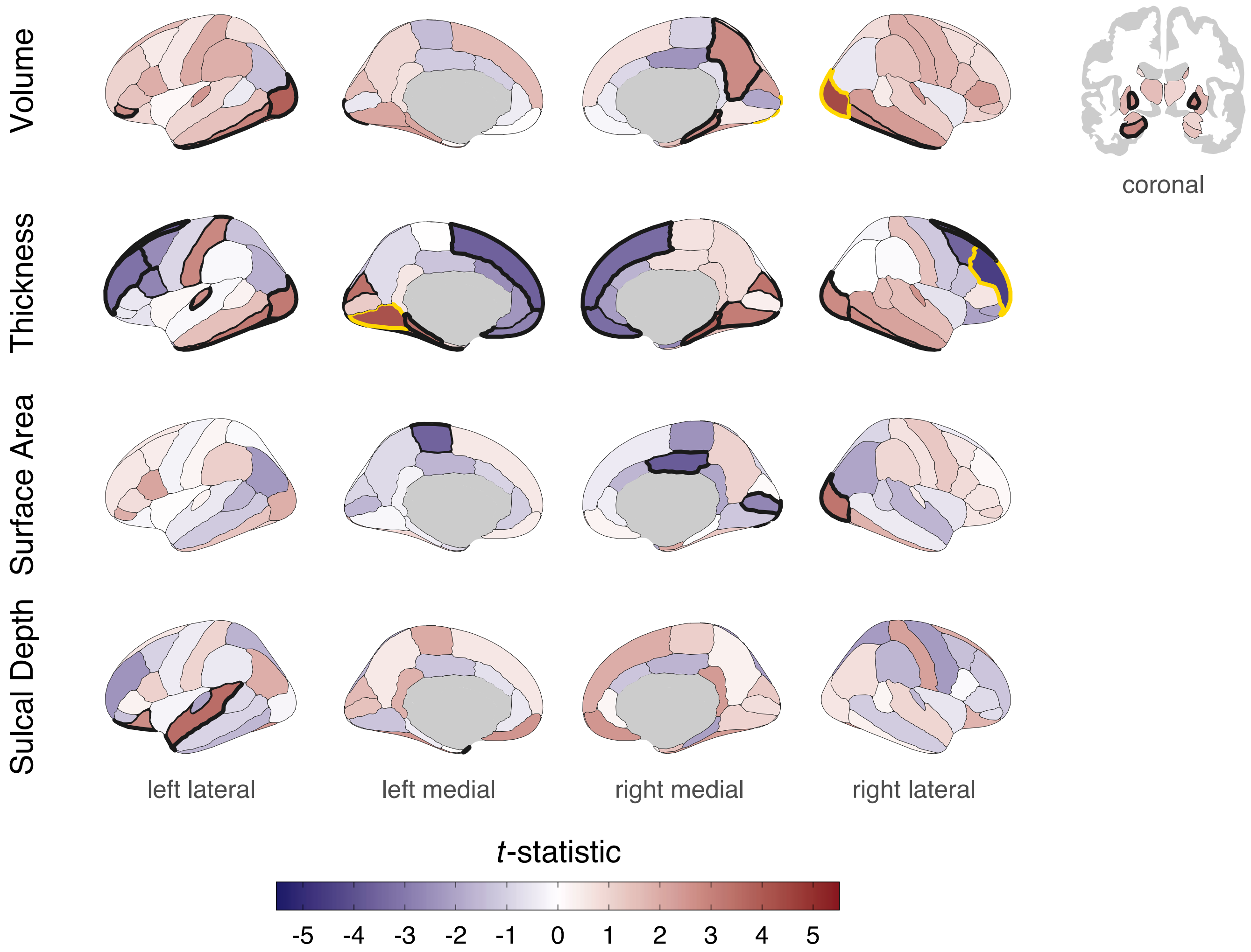

eFigure 3. Regional cortical and subcortical associations with any early substance use initiation in ABCD. Regional brain plots were constructed using the *ggseg* package in R.[46] Cortical and subcortical patterning of associations with any substance use initiation are plotted as *t*-statistics (red = positive association, blue = negative association) according to the Desikan–Killiany cortical atlas[47] and the Automatic Segmentation of Subcortical Structures atlas,[48] respectively. Regions with bold yellow outlines were Bonferroni-significant for all study comparisons, and those with bold black outlines exhibited FDR-significant associations. Bonferroni-significant regions: volume of R lateral occipital; thickness of R rostral middle frontal and L lingual. FDR-significant regions: volume of R/L globus pallidus and inferior temporal, and R parahippocampal and precuneus, and L hippocampus, lateral occipital, and pars orbitalis; thickness of R/L cuneus, inferior temporal, lateral occipital, medial orbitofrontal, parahippocampal, and superior frontal, and R caudal middle frontal, frontal pole, and lingual, and L fusiform, pars opercularis, postcentral, rostral middle frontal, and transverse temporal; surface area of R lateral occipital, pericalcarine, and posterior cingulate, and L paracentral; sulcal depth of L superior temporal and temporal pole (see also **eTable 3 in Supplement 1**).

© 2024 Miler AP et al. *JAMA Network Open.*

eFigure 4. Regional cortical and subcortical associations with early alcohol, nicotine, and cannabis use initiation in ABCD.

Regional brain plots were constructed using the *ggseg* package in R.[46] Cortical and subcortical patterning of associations with initiation of alcohol, cannabis, and nicotine are plotted as *t*-statistics (red = positive association, blue = negative association) according to the Desikan–Killiany cortical atlas[47] and the Automatic Segmentation of Subcortical Structures atlas,[48] respectively. Regions with bold yellow outlines were Bonferroni-significant for all study comparisons, and those with bold black outlines exhibited FDR-significant associations. For **alcohol (A)**, Bonferroni-significant regions include volume of R/L lateral occipital; thickness of R/L parahippocampal, R/L superior frontal, R rostral middle frontal, and L lingual, and FDR-significant regions include volume of R/L inferior temporal, R parahippocampal, and L globus pallidus, hippocampus, and pars orbitalis; thickness of R/L cuneus, inferior temporal, and lateral occipital, R caudal middle frontal, lingual, and medial orbitofrontal, and L fusiform, pars opercularis, rostral middle frontal, and transverse temporal; surface area of R lateral occipital and posterior cingulate, and L paracentral; sulcal depth of L superior temporal and temporal pole (see also **eTable 5 in Supplement 1**). For **nicotine (B)**, FDR-significant associations include volume of R superior frontal and sulcal depth of L lateral orbitofrontal (see also **eTable 7 in Supplement 1**). For **cannabis (C)**, FDR-significant associations include volume of R inferior parietal and caudate and thickness of L precentral (see also **eTable 9 in Supplement 1**).

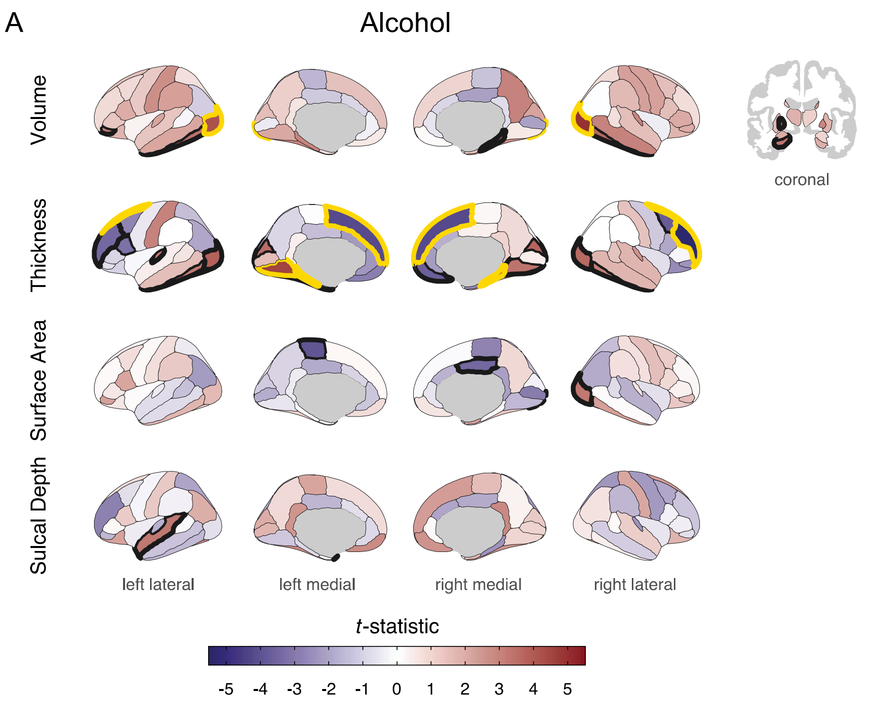

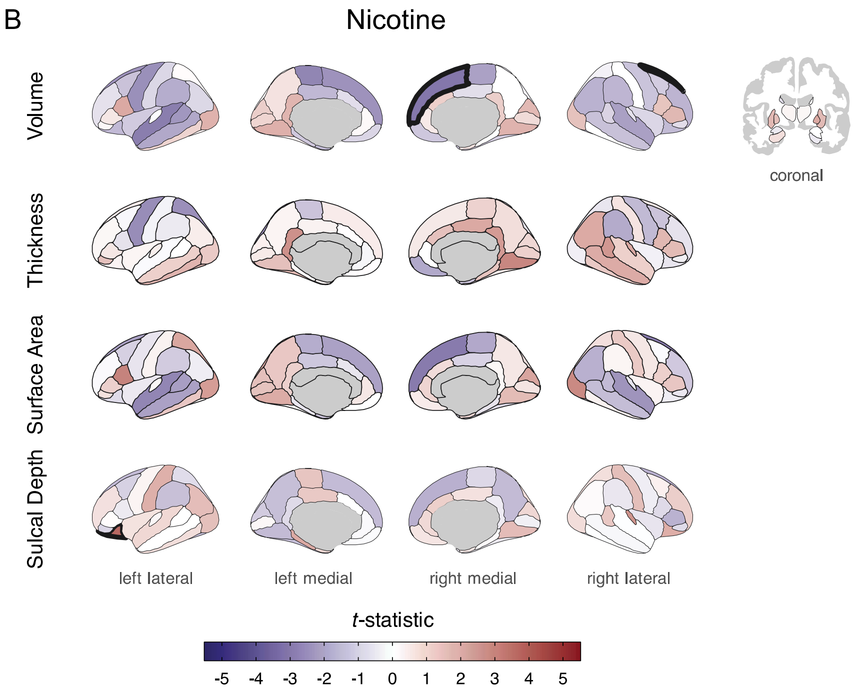

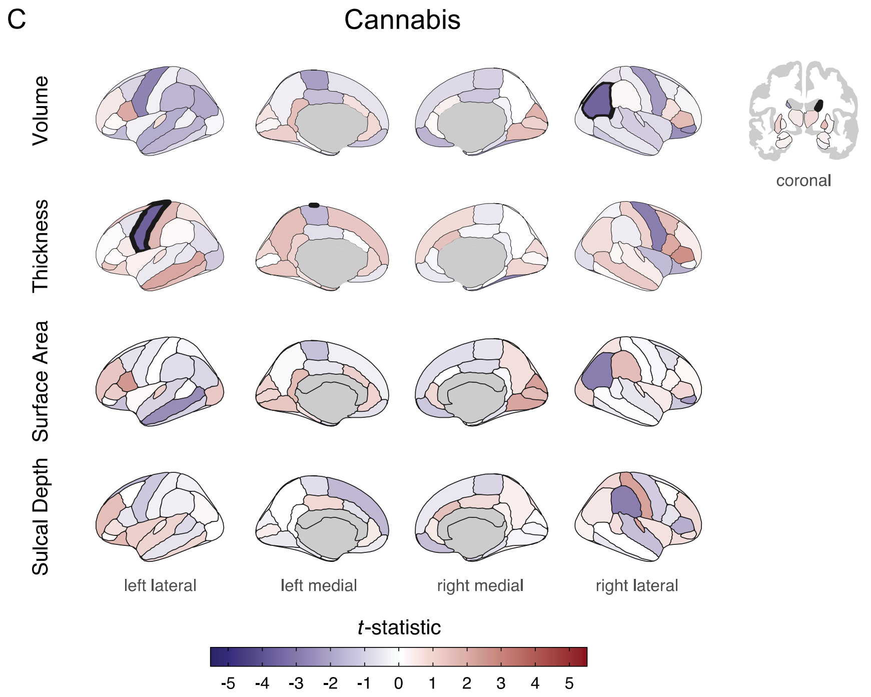

© 2024 Miler AP et al. *JAMA Network Open.*

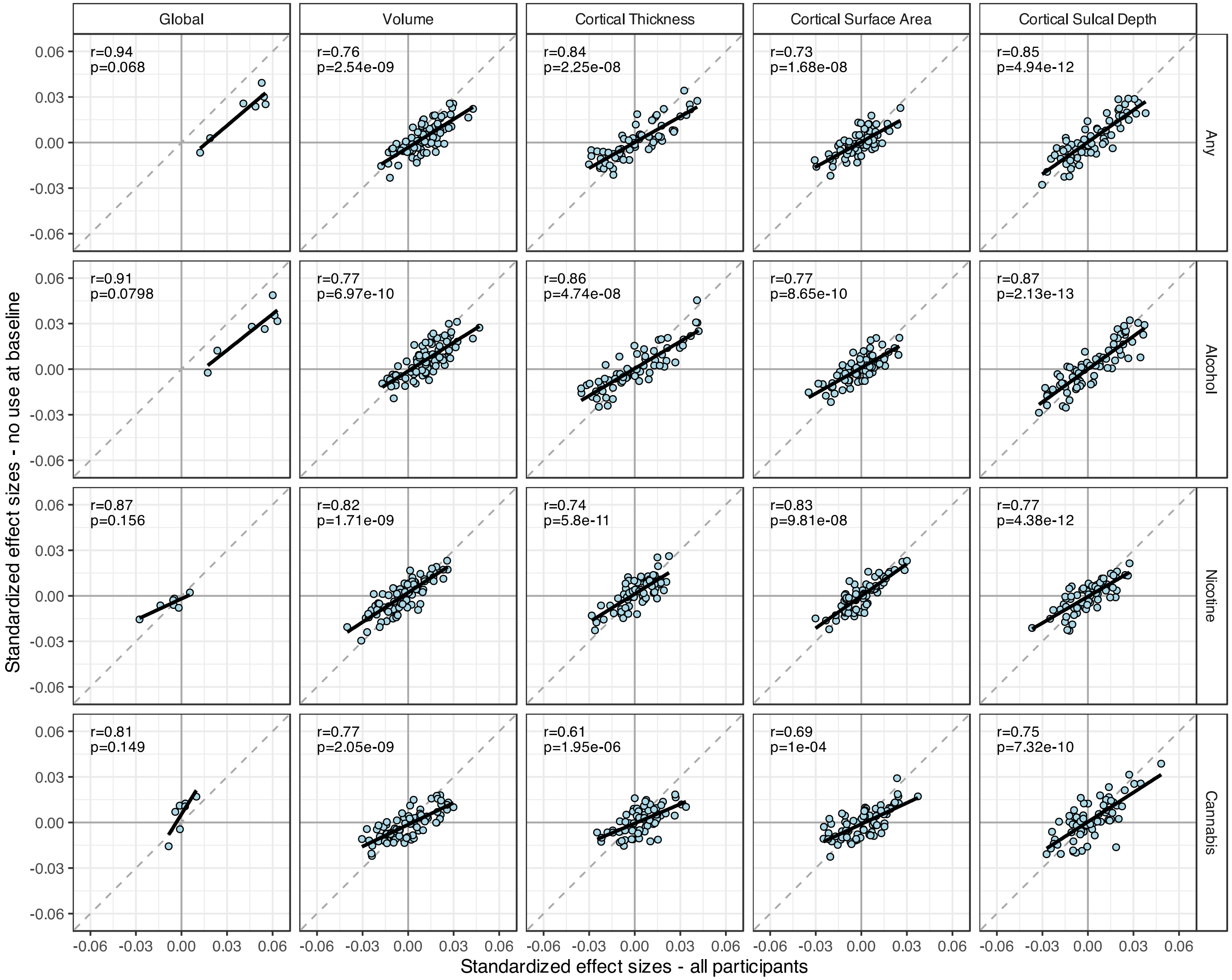

eFigure 5. **Correlations between effect sizes in analyses in the full sample and analyses restricted to participants without baseline substance use initiation.** Exchangeability block permutations with accelerated *P*-values were used to determine the significance of the correlations. Results show a high correspondence in effect sizes between the two analyses.

© 2024 Miler AP et al. *JAMA Network Open.*

eReferences.

1. Watts AL, Doss MI, Bernard DL, Sher KJ. Psychopathology as dynamic markers of alcohol initiation across development: A three-year longitudinal examination. *Dev Psychopathol*. Published online March 20, 2023:1-10. doi:10.1017/S0954579423000184
2. Watts AL, Wood PK, Jackson KM, et al. Incipient alcohol use in childhood: Early alcohol sipping and its relations with psychopathology and personality. *Dev Psychopathol*. 2021;33(4):1338-1350. doi:10.1017/S0954579420000541
3. Casey BJ, Cannonier T, Conley MI, et al. The Adolescent Brain Cognitive Development (ABCD) study: Imaging acquisition across 21 sites. *Dev Cogn Neurosci*. 2018;32:43-54. doi:10.1016/j.dcn.2018.03.001
4. Hagler DJ, Hatton S, Cornejo MD, et al. Image processing and analysis methods for the Adolescent Brain Cognitive Development Study. *NeuroImage*. 2019;202:116091. doi:10.1016/j.neuroimage.2019.116091
5. Ségonne F, Dale AM, Busa E, et al. A hybrid approach to the skull stripping problem in MRI. *NeuroImage*. 2004;22(3):1060-1075. doi:10.1016/j.neuroimage.2004.03.032
6. Fischl B, Sereno MI, Dale AM. Cortical surface-based analysis. II: Inflation, flattening, and a surface-based coordinate system. *NeuroImage*. 1999;9(2):195-207. doi:10.1006/nimg.1998.0396
7. Yang X, Tian F, Zhang H, et al. Cortical and subcortical gray matter shrinkage in alcohol-use disorders: a voxel-based meta-analysis. *Neurosci Biobehav Rev*. 2016;66:92-103. doi:10.1016/j.neubiorev.2016.03.034
8. Tadayon E, Pascual-Leone A, Santarnecchi E. Differential Contribution of Cortical Thickness, Surface Area, and Gyrification to Fluid and Crystallized Intelligence. *Cereb Cortex N Y NY*. 2020;30(1):215-225. doi:10.1093/cercor/bhz082
9. Alcover KC, Thompson CL. Patterns of Mean Age at Drug Use Initiation Among Adolescents and Emerging Adults, 2004-2017. *JAMA Pediatr*. 2020;174(7):725-727. doi:10.1001/jamapediatrics.2019.6235
10. Fuhrmann D, Knoll LJ, Blakemore SJ. Adolescence as a Sensitive Period of Brain Development. *Trends Cogn Sci*. 2015;19(10):558-566. doi:10.1016/j.tics.2015.07.008
11. Kuhn C. Emergence of sex differences in the development of substance use and abuse during adolescence. *Pharmacol Ther*. 2015;153:55-78. doi:10.1016/j.pharmthera.2015.06.003
12. Lenroot RK, Giedd JN. Sex differences in the adolescent brain. *Brain Cogn*. 2010;72(1):46-55. doi:10.1016/j.bandc.2009.10.008
13. Castellanos-Ryan N, Parent S, Vitaro F, Tremblay RE, Séguin JR. Pubertal Development, Personality, and Substance Use: A 10-Year Longitudinal Study From Childhood to Adolescence. *J Abnorm Psychol*. 2013;122(3):782-796. doi:10.1037/a0033133
14. Vijayakumar N, Op de Macks Z, Shirtcliff EA, Pfeifer JH. Puberty and the human brain: Insights into adolescent development. *Neurosci Biobehav Rev*. 2018;92:417-436. doi:10.1016/j.neubiorev.2018.06.004
15. Westling E, Andrews JA, Hampson SE, Peterson M. Pubertal Timing and Substance Use: The Effects of Gender, Parental Monitoring and Deviant Peers. *J Adolesc Health*. 2008;42(6):555-563. doi:10.1016/j.jadohealth.2007.11.002
16. Jansen AG, Mous SE, White T, Posthuma D, Polderman TJC. What Twin Studies Tell Us About the Heritability of Brain Development, Morphology, and Function: A Review. *Neuropsychol Rev*. 2015;25(1):27-46. doi:10.1007/s11065-015-9278-9
17. Neiderhiser JM, Marceau K, Reiss D. Four factors for the initiation of substance use by young adulthood: A 10-year follow-up twin and sibling study of marital conflict, monitoring, siblings, and peers. *Dev Psychopathol*. 2013;25(1):133-149. doi:10.1017/S0954579412000958
18. Han X, Jovicich J, Salat D, et al. Reliability of MRI-derived measurements of human cerebral cortical thickness: The effects of field strength, scanner upgrade and manufacturer. *NeuroImage*. 2006;32(1):180-194. doi:10.1016/j.neuroimage.2006.02.051
19. Takao H, Hayashi N, Ohtomo K. Effects of study design in multi-scanner voxel-based morphometry studies. *NeuroImage*. 2014;84:133-140. doi:10.1016/j.neuroimage.2013.08.046
20. Petersen AC, Crockett L, Richards M, Boxer A. A self-report measure of pubertal status: Reliability, validity, and initial norms. *J Youth Adolesc*. 1988;17(2):117-133. doi:10.1007/BF01537962
21. Owens MM, Allgaier N, Hahn S, et al. Multimethod investigation of the neurobiological basis of ADHD symptomatology in children aged 9-10: baseline data from the ABCD study. *Transl Psychiatry*. 2021;11:64. doi:10.1038/s41398-020-01192-8
22. Petrican R, Miles S, Rudd L, Wasiewska W, Graham KS, Lawrence AD. Pubertal timing and functional neurodevelopmental alterations independently mediate the effect of family conflict on adolescent psychopathology. *Dev Cogn Neurosci*. 2021;52:101032. doi:10.1016/j.dcn.2021.101032

© 2024 Miler AP et al. *JAMA Network Open.*

23. Thijssen S, Collins PF, Luciana M. Pubertal development mediates the association between family environment and brain structure and function in childhood. *Dev Psychopathol*. 2020;32(2):687-702. doi:10.1017/S0954579419000580
24. Baranger DA, Miller AP, Gorelik AJ, et al. Prenatal cannabis exposure is associated with localized brain differences that partially mediate associations with increased adolescent psychopathology. *medRxiv*. Published online October 17, 2023:2023.09.19.23295792. doi:10.1101/2023.09.19.23295792
25. Fortin JP, Cullen N, Sheline YI, et al. Harmonization of cortical thickness measurements across scanners and sites. *NeuroImage*. 2018;167:104-120. doi:10.1016/j.neuroimage.2017.11.024
26. Dumornay NM, Lebois LAM, Ressler KJ, Harnett NG. Racial Disparities in Adversity During Childhood and the False Appearance of Race-Related Differences in Brain Structure. *Am J Psychiatry*. 2023;180(2):127-138. doi:10.1176/appi.ajp.21090961
27. Noble KG, Houston SM, Kan E, Sowell ER. Neural correlates of socioeconomic status in the developing human brain. *Dev Sci*. 2012;15(4):516-527. doi:10.1111/j.1467-7687.2012.01147.x
28. Rakesh D, Whittle S. Socioeconomic status and the developing brain – A systematic review of neuroimaging findings in youth. *Neurosci Biobehav Rev*. 2021;130:379-407. doi:10.1016/j.neubiorev.2021.08.027
29. Lee JO, Jones TM, Kosterman R, et al. Childhood neighborhood context and adult substance use problems: the role of socio-economic status at the age of 30 years. *Public Health*. 2018;165:58-66. doi:10.1016/j.puhe.2018.09.011
30. Patrick ME, Wightman P, Schoeni RF, Schulenberg JE. Socioeconomic Status and Substance Use Among Young Adults: A Comparison Across Constructs and Drugs. *J Stud Alcohol Drugs*. 2012;73(5):772-782. doi:10.15288/jsad.2012.73.772
31. Zapolski TCB, Pedersen SL, McCarthy DM, Smith GT. Less drinking, yet more problems: understanding African American drinking and related problems. *Psychol Bull*. 2014;140(1):188-223. doi:10.1037/a0032113
32. Koob GF, Volkow ND. Neurobiology of addiction: a neurocircuitry analysis. *Lancet Psychiatry*. 2016;3(8):760-773. doi:10.1016/S2215-0366(16)00104-8
33. Bogdan R, Hatoum AS, Johnson EC, Agrawal A. The Genetically Informed Neurobiology of Addiction (GINA) model. *Nat Rev Neurosci*. 2023;24(1):40-57. doi:10.1038/s41583-022-00656-8
34. Baranger DAA, Paul SE, Hatoum AS, Bogdan R. Alcohol use and grey matter structure: Disentangling predispositional and causal contributions in human studies. *Addict Biol*. 2023;28(9):e13327. doi:10.1111/adb.13327
35. Mackey S, Allgaier N, Chaarani B, et al. Mega-Analysis of Gray Matter Volume in Substance Dependence: General and Substance-Specific Regional Effects. *Am J Psychiatry*. 2019;176(2):119-128. doi:10.1176/appi.ajp.2018.17040415
36. Lorenzetti V, Chye Y, Silva P, Solowij N, Roberts CA. Does regular cannabis use affect neuroanatomy? An updated systematic review and meta-analysis of structural neuroimaging studies. *Eur Arch Psychiatry Clin Neurosci*. 2019;269(1):59-71. doi:10.1007/s00406-019-00979-1
37. Pando-Naude V, Toxto S, Fernandez-Lozano S, Parsons CE, Alcauter S, Garza-Villarreal EA. Gray and white matter morphology in substance use disorders: a neuroimaging systematic review and meta-analysis. *Transl Psychiatry*. 2021;11(1):1-18. doi:10.1038/s41398-020-01128-2
38. Yang Z, Zhang Y, Cheng J, Zheng R. Meta-analysis of brain gray matter changes in chronic smokers. *Eur J Radiol*. 2020;132:109300. doi:10.1016/j.ejrad.2020.109300
39. Owens MM, Hyatt CS, Gray JC, et al. Neuroanatomical correlates of impulsive traits in children aged 9 to 10. *J Abnorm Psychol*. 2020;129(8):831-844. doi:10.1037/abn0000627
40. Bernanke J, Luna A, Chang L, Bruno E, Dworkin J, Posner J. Structural brain measures among children with and without ADHD in the Adolescent Brain and Cognitive Development Study cohort: a cross-sectional US population-based study. *Lancet Psychiatry*. 2022;9(3):222-231. doi:10.1016/S2215-0366(21)00505-8
41. Shen X, MacSweeney N, Chan SWY, et al. Brain structural associations with depression in a large early adolescent sample (the ABCD study®). *eClinicalMedicine*. 2021;42. doi:10.1016/j.eclinm.2021.101204
42. Markello RD, Misic B. Comparing spatial null models for brain maps. *NeuroImage*. 2021;236:118052. doi:10.1016/j.neuroimage.2021.118052
43. Winkler AM, Webster MA, Vidaurre D, Nichols TE, Smith SM. Multi-level block permutation. *NeuroImage*. 2015;123:253-268. doi:10.1016/j.neuroimage.2015.05.092
44. Winkler AM, Ridgway GR, Douaud G, Nichols TE, Smith SM. Faster permutation inference in brain imaging. *NeuroImage*. 2016;141:502-516. doi:10.1016/j.neuroimage.2016.05.068
45. Delignette-Muller ML, Dutang C. fitdistrplus: An R Package for Fitting Distributions. *J Stat Softw*. 2015;64:1-34. doi:10.18637/jss.v064.i04

© 2024 Miler AP et al. *JAMA Network Open.*

46. Mowinckel AM, Vidal-Piñeiro D. Visualization of Brain Statistics With R Packages ggseg and ggseg3d. *Adv Methods Pract Psychol Sci.* 2020;3(4):466-483. doi:10.1177/2515245920928009
47. Desikan RS, Ségonne F, Fischl B, et al. An automated labeling system for subdividing the human cerebral cortex on MRI scans into gyral based regions of interest. *NeuroImage*. 2006;31(3):968-980. doi:10.1016/j.neuroimage.2006.01.021
48. Fischl B, Salat DH, Busa E, et al. Whole brain segmentation: automated labeling of neuroanatomical structures in the human brain. *Neuron*. 2002;33(3):341-355. doi:10.1016/s0896-6273(02)00569-x

© 2024 Miler AP et al. *JAMA Network Open.*
