# Supplementary material for: Neuroanatomical Variability and Substance Use Initiation in Late Childhood and Early Adolescence
Source: JAMA Netw Open. 2024 Dec 30;7(12):e2452027. doi: 10.1001/jamanetworkopen.2024.52027 (PMC11686416; doi:10.1001/jamanetworkopen.2024.52027)
Supplement: Supplement 3. — Data Sharing Statement [file jamanetwopen-e2452027-s003.pdf]

Data Sharing Statement

Data
Data available: Yes
Data types: Deidentified participant data, Data dictionary
How to access data: The ABCD data repository grows and changes over time. The ABCD Data repository and codebook are available via the ABCD Wiki (https://wiki.abcdstudy.org/). The ABCD data used in this report came from http://dx.doi.org/10.15154/1520591 (ABCD Annual Release 3.0) and http://dx.doi.org/10.15154/8873-zj65 (ABCD Annual Release 5.0). DOIs can be found at https://nda.nih.gov/abcd/abcd-annual-releases.html.
When available: With publication

Supporting Documents
Document types: None

Additional Information
Who can access the data: Qualified researchers can request access to ABCD shared data.
Types of analyses: Analyses detailed in access proposal and objectively and systematically reviewed the ABCD data access committee (DAC).
Mechanisms of data availability: Mechanisms for requesting access to ABCD shared data are detailed here: https://nda.nih.gov/abcd/request-access.html
